# Healthcare Worker Occupation and Immune Response to *Pneumocystis jirovecii*

**DOI:** 10.3201/eid1510.090207

**Published:** 2009-10

**Authors:** Renuka Tipirneni, Kieran R. Daly, Leah G. Jarlsberg, Judy V. Koch, Alexandra Swartzman, Brenna M. Roth, Peter D. Walzer, Laurence Huang

**Affiliations:** San Francisco General Hospital/University of California, San Francisco, California, USA (R. Tipirneni, L.G. Jarlsberg, A. Swartzman, B.M. Roth, L. Huang); Veterans Affairs Medical Center/University of Cincinnati, Cincinnati, Ohio, USA (K.R. Daly, J.V. Koch, P.D. Walzer)

**Keywords:** Pneumocystis, health personnel, HIV/AIDS and other retroviruses, opportunistic infections, antibodies, fungal, fungi, serologic tests, research

## Abstract

The reservoir and mode of transmission of *Pneumocystis jirovecii* remain uncertain. We conducted a cross-sectional study of 126 San Francisco General Hospital staff in clinical (n = 103) and nonclinical (n = 23) occupations to assess whether occupational exposure was associated with immune responses to *P. jirovecii*. We examined antibody levels by ELISA for 3 overlapping fragments that span the *P. jirovecii* major surface glycoprotein (Msg): MsgA, MsgB, and MsgC1. Clinical occupation participants had higher geometric mean antibody levels to MsgC1 than did nonclinical occupation participants (21.1 vs. 8.2, p = 0.004); clinical occupation was an independent predictor of higher MsgC1 antibody levels (parameter estimate = 0.89, 95% confidence interval 0.29–1.48, p = 0.003). In contrast, occupation was not significantly associated with antibody responses to either MsgA or MsgB. Healthcare workers may have occupational exposure to *P. jirovecii*. Humans may be a reservoir for *P. jirovecii* and may transmit it from person to person.

## Abstract 2

Humans may be a reservoir for this pathogen and transmit it from person to person.

Although the incidence of *Pneumocystis* *jirovecii* pneumonia (PCP) has declined in the era of combination antiretroviral therapy, PCP remains the most common serious opportunistic infection among human immunodeficiency virus (HIV)-infected persons in the United States (*1*). The reservoir and mode of transmission of *P. jirovecii* remain uncertain because of an inability to grow the organism in vitro. However, studies of immune responses to *P. jirovecii* have provided important insights into its epidemiology, showing that up to 80%–100% of children have detectable *P.* *jirovecii* antibodies by 8 years of age (*2*–*9*). These findings suggest that *P. jirovecii* is ubiquitous, that humans are exposed to *P. jirovecii* early in life, and that PCP that develops later in life results from reactivation of latent infection.

Emerging evidence suggests that PCP also can result from recent acquisition of *P.* *jirovecii*, and the organism may be transmitted from person to person (*10**,**11*). In the hospital or clinic, numerous PCP outbreaks have been reported among immunocompromised patients who shared common healthcare workers (HCWs), hospital rooms, wards, or clinics (*12*–*21*). In the laboratory, animal-to-animal transmission of *Pneumocystis* spp. has been demonstrated both by immunocompromised and immunocompetent hosts after periods of exposure as short as 1 day (*22**,**23**)*. Dumoulin et al. demonstrated that immunocompetent mice became transiently colonized with *Pneumocystis* spp. after contact with *Pneumocystis*-infected mice and then were able to transmit the infection to *Pneumocystis*-free mice that had severe combined immunodeficiency (*23*).

Several studies have found that *P. jirovecii* can colonize immunocompetent humans and suggest that such persons may serve as potential reservoirs (*24*). The question that arises is whether person-to-person transmission occurs through immunocompetent hosts, such as HCWs, who may be transiently colonized with *P. jirovecii* during brief clinical interactions with PCP patients and subsequently transmit the infection to other immunocompromised patients. Prior studies involving HCWs used different specimens (e.g., induced sputum, oropharyngeal wash, nasal rinse, deep nasal swab, blood) and different laboratory methods (i.e., different PCR and ELISA) to compare exposed and unexposed groups, making findings difficult to compare across studies (*25*–*31*). In addition, these studies compared different groups of HCWs and did not include a control group without patient contact.

Therefore, we performed a cross-sectional study of hospital staff at San Francisco General Hospital (SFGH) in both clinical (exposed) and nonclinical (unexposed) occupations. Our goal was to determine whether HCW occupation was associated with antibody levels to *P. jirovecii*. Finding this association would suggest that HCWs may acquire *P. jirovecii* and potentially be a reservoir in the hospital setting.

## Methods

### Participants

We recruited participants from the Department of Medicine, the Division of Pulmonary and Critical Care Medicine, and the HIV/AIDS Division because members of these groups provide the most care to patients with HIV infection or PCP, our hypothesized primary reservoirs of *P. jirovecii*. We recruited by word of mouth; emails to departmental listservs; and announcements at medical conferences, staff meetings, and orientations for medical students and residents. From January 2007 through February 2008, we enrolled 126 SFGH staff. We included staff who worked at the hospital during the study period, provided informed consent, and had no clinical evidence of PCP. The University of California, San Francisco, and the University of Cincinnati institutional review boards approved the study.

### Questionnaire

We collected information by using a standardized participant-completed questionnaire. In addition to demographic characteristics, we obtained information about occupation, department/division, percentage of time spent seeing patients, and past exposure to patients with PCP. These questions were designed to assess patient contact in general and contact with PCP patients specifically. We also obtained information about cigarette smoking, chronic lung disease (e.g., chronic obstructive pulmonary disease), and immunocompromising conditions (e.g., HIV infection or use of immunosuppressive medications) because these factors have been associated with *P. jirovecii* colonization (*24*). To protect our colleagues’ confidentiality regarding their medical history, we asked participants to check yes if they had >1 condition from a list of pulmonary or immunocompromising conditions, without requiring them to specify the condition. For example, participants were asked to reply yes or no to the following question: “Do you have any of the following conditions? HIV, cancer including leukemia/lymphoma, organ transplant, bone marrow transplant, steroid medication (e.g., prednisone), chemotherapy medication, immunosuppressive medication (e.g., methotrexate, rituximab, cyclosporine, tacrolimus, azathioprine, cyclophosphamide), pregnancy,” but participants were not required to disclose the specific immune disorder (e.g., HIV infection, cancer).

### Classification of Participants

We classified staff into 2 groups: those with patient contact (clinical occupation group, n = 103) and those without contact with patients or clinical samples from patients (nonclinical occupation group, n = 23). The clinical group was further subdivided into staff who provide direct patient care (e.g., physicians and nurses) and staff who provide indirect patient care or ancillary services (e.g., medical assistants, social workers, and pharmacists). Because each department/division has defined space on the SFGH campus, people from the same department/division generally have offices in the same location. However, those in the clinical occupation group also work in the hospital wards, clinics, or clinical research units and have contact with patients, whereas those in the nonclinical group have no occupational contact with patients.

### Serum ELISA

**Figure 1:**
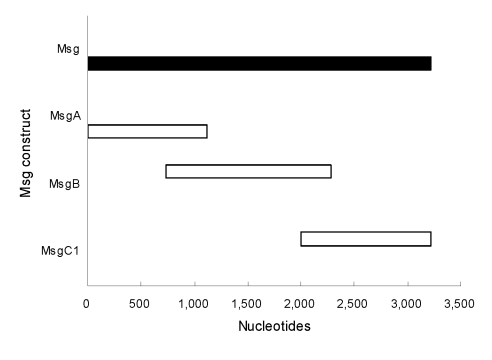
*Pneumocystis* *jirovecii* major surface glycoprotein (Msg) fragments. Lengths of Msg fragments are expressed on a nucleotide scale. MsgA is the amino terminus, MsgB is the middle portion, and MsgC1 is the carboxyl terminus of the protein.

We collected a serum specimen from each participant and stored it at −80ºC before shipping it to the University of Cincinnati. We used a previously described ELISA (*32*,*33*) to measure immunoglobulin G levels to 3 overlapping fragments that span the length of the *P. jirovecii* major surface glycoprotein (Msg): MsgA, MsgB, and MsgC1 (Figure 1). MsgA is the amino terminus, MsgB is the middle portion, and MsgC is the carboxyl terminus (*34*). MsgC1 is 1 of 4 MsgC variants we have generated and can be used to distinguish between HIV-infected patients with and without PCP on the basis of antigenic recognition (*33*). We tested participant serum specimens and the reference serum against Msg fragments and used phosphate-buffered saline as a negative control. We corrected the reactivity of each serum specimen to Msg by subtracting the reactivity of that serum to phosphate-buffered saline and quantified the results by using the method of Bishop and Kovacs (*35*). A standard curve generated for each Msg construct was used to calculate the units of reactivity. We assigned to each standard serum pool a value of 100 U of reactivity to its target Msg construct in 100 µL of a 1:100 dilution. We assayed test specimens at 1:100–1:200 dilutions to fit the linear portion of the standard curves and calculated units of reactivity. Samples with values below the standard curve were assigned the lowest possible value of 1 U.

### Statistical Analysis

The laboratory group was blinded to the clinical data, and the clinical group was blinded to the laboratory results. Prespecified primary predictors of interest were professional and health characteristics. The outcome of interest was Msg antibody level, a continuous variable, which we log transformed to better approximate a normal distribution. We calculated the mean log Msg level for each predictor variable and examined bivariate associations using Student *t* test. The data were then converted to the original scale and presented as geometric means (GMs). For associations with p<0.1 in bivariate analysis, we performed multivariate linear regression using the natural log of Msg level as the dependent variable and considered a 2-tailed p<0.05 to be statistically significant. All statistics were calculated using SAS software, version 9.1 (SAS Institute Inc., Cary, NC, USA).

## Results

### Participants

We enrolled 126 staff. Mean age of participants was 39.6 years (range 22–80 years), 57.1% were female, 60.2% were white/Caucasian, 25.2% were Asian, 16.0% were Hispanic/Latino, and 3.3% were black/African American (Table 1). Forty-two (33.6%) had smoked at least 100 cigarettes in their lifetime, and 16.0% had chronic lung disease, including asthma (n = 17) and interstitial lung disease (n = 1). Overall, 6.4% had an immunocompromising condition. Participants were primarily from the HIV/AIDS Division (44.4%), the Division of Pulmonary and Critical Care Medicine (26.2%), and the Department of Medicine (23.0%). A few participants (6.4%) were from other departments (Obstetrics and Gynecology, Psychiatry, and Radiology) and were involved in the care of HIV-infected or PCP patients. Eighty-five (67.5%) participants reported prior exposure to a PCP patient.

**Table 1:** Characteristics of participants in a study of *Pneumocystis jirovecii* antibody levels*

Characteristic	Total, no. (%), N = 126	Clinical, no. (%), n = 103	Nonclinical, no. (%), n = 23	p value
Demographics				
Age group, y				
<30	33 (26.6)	30 (29.7)	3 (13.0)	Ref
31–40	38 (30.7)	31 (30.7)	7 (30.4)	0.98
41–50	27 (21.8)	20 (19.8)	7 (30.4)	0.35
51–60	21 (16.9)	16 (15.8)	5 (21.7)	0.53
>60	5 (4.0)	4 (4.0)	1 (4.4)	0.92
Sex				
F	72 (57.1)	57 (55.3)	15 (65.2)	0.39
M	54 (42.9)	46 (44.7)	8 (34.8)	Ref
Race				
White/Caucasian	74 (60.2)	60 (60.0)	14 (60.9)	Ref
Asian	31 (25.2)	27 (27.0)	4 (17.4)	0.14
Black/African American	4 (3.3)	2 (2.0)	2 (8.7)	0.14
Other	14 (11.4)	11 (11.0)	3 (13.0)	0.82
Hispanic/Latino ethnicity	20 (16.0)	14 (13.7)	6 (26.1)	0.20
Health conditions				
Ever smoked	42 (33.6)	32 (31.4)	10 (43.5)	0.27
Lung condition	20 (16.0)	14 (13.7)	6 (26.1)	0.20
Immune condition	8 (6.4)	4 (3.9)	4 (17.4)	0.04
Professional characteristics				
Department/division				
HIV/AIDS	56 (44.4)	45 (43.7)	11 (47.8)	Ref
Pulmonary and CCM	33 (26.2)	28 (27.2)	5 (21.7)	0.42
Medicine	29 (23.0)	25 (24.3)	4 (17.4)	0.32
Other	8 (6.4)	5 (4.9)	3 (13.0)	0.14
Ever exposed to PCP patient	85 (67.5)	83 (80.6)	2 (8.7)	<0.001

*Ref, reference category; CCM, Critical Care Medicine; PCP, *P.* *jirovecii* pneumonia. Participants in this cross-sectional study were healthcare staff recruited during January 2007–February 2008 from San Francisco General Hospital. Mean ages (y ± SD) of participants: total, 39.6 ± 11.7; clinical, 39.1 ± 11.9; nonclinical, 42.0 ± 10.4 (p = 0.29).

### Clinical and Nonclinical Occupation Groups

We classified 103 (81.7%) participants into the clinical occupation group and 23 (18.3%) into the nonclinical occupation group. The clinical group consisted of 27 attending physicians; 17 residents and fellows; 19 medical students; 9 nurse practitioners; 10 nurses; 10 ancillary clinic staff, including medical assistants, social workers, pharmacists, and clinic managers; and 11 clinical research personnel. The nonclinical group consisted of 18 administrative staff and 5 laboratory personnel. These 2 groups did not differ significantly in terms of demographic characteristics (Table 1). However, a significantly greater proportion of the nonclinical group than the clinical group reported having an immunocompromising condition (17.4% vs. 3.9%, p = 0.04), and a significantly greater proportion of the clinical group than the nonclinical group reported prior exposure to a PCP patient (83 [80.6%] of 103 vs. 2 [8.7%] of 23, p<0.001).

### Antibody Levels to MsgA, MsgB, and MsgC1

Participants had detectable antibody levels to MsgA (GM 11.8, 95% CI 8.1–17.0), MsgB (GM 2.6, 95% confidence interval [CI] 2.1–3.1), and MsgC1 (GM 17.8, 95% CI 13.8–22.9) (Table 2). Antibody responses were detected in participants from all demographic groups, in smokers and nonsmokers, and in participants with and without chronic lung disease or immunocompromising condition. Responses also were detected in participants with and without exposure to a PCP patient and in both the clinical and nonclinical groups.

**Table 2:** Bivariate associations with *Pneumocystis* *jirovecii* Msg levels*

Characteristic	No. persons	MsgA			MsgB			MsgC1	
		GM (95% CI)	p value		GM (95% CI)	p value		GM (95% CI)	p value
Total	126	11.8 (8.1–17.0)			2.6 (2.1–3.1)			17.8 (13.8–22.9)	
Demographics									
Age group, y									
<30	33	12.0 (5.4–26.8)	Ref		2.5 (1.7–3.7)	Ref		16.3 (9.5–28.0)	Ref
31–40	38	11.0 (5.7–21.3)	0.86		2.7 (1.8–3.9)	0.84		22.6 (14.9–34.4)	0.33
41–50	27	10.1 (4.5–22.4)	0.75		2.4 (1.6–3.7)	0.88		19.8 (11.6–33.7)	0.61
51–60	21	17.2 (6.3–46.4)	0.57		2.4 (1.4–4.2)	0.90		17.4 (8.8–34.3)	0.88
>60	5	12.8 (0.7–243.0)	0.95		3.3 (0.3–32.8)	0.66		4.8 (0.7–34.7)	0.11
Sex									
M	54	14.3 (8.2–25.2)	0.37		2.9 (2.1–4.0)	0.26		22.7 (16.0–32.1)	0.10
F	72	10.2 (6.2–16.7)			2.3 (1.8–3.0)			14.8 (10.4–21.2)	
Race									
Asian	31	6.6 (3.1–14.4)	0.08		2.3 (1.6–3.3)	0.49		27.5 (17.6–42.9)	0.05
Other	92	14.4 (9.4–22.1)			2.7 (2.1–3.5)			15.3 (11.2–20.7)	
Ethnicity									
Hispanic/Latino	20	12.5 (5.5–28.5)	0.92		1.9 (1.3–2.7)	0.17		13.4 (6.2–29.1)	0.35
Non–Hispanic/Latino	105	11.9 (7.9–18.1)			2.7 (2.2–3.5)			18.7 (14.2–24.4)	
Health conditions									
Smoked									
Ever	42	13.9 (7.1–27.1)	0.58		2.8 (1.9–4.1)	0.63		13.2 (8.4–20.9)	0.11
Never	83	11.2 (7.1–17.5)			2.5 (2.0–3.2)			20.5 (15.1–27.8)	
Lung condition									
Yes	20	11.3 (4.4–28.7)	0.88		2.5 (1.5–4.1)	0.83		14.1 (7.2–27.5)	0.45
No	105	12.2 (8.1–18.3)			2.6 (2.1–3.3)			18.5 (14.0–24.4)	
Immune condition									
Yes	8	13.1 (2.6–66.6)	0.90		2.9 (0.8–10.0)	0.76		24.0 (13.6–42.3)	0.26
No	117	11.9 (8.1–17.6)			2.6 (2.1–3.1)			17.3 (13.2–22.7)	
Professional characteristics									
Exposed to PCP patient									
Ever	85	13.1 (8.3–20.7)	0.41		2.6 (2.0–3.3)	0.99		20.8 (15.8–27.3)	0.11
Never	41	9.4 (4.9–18.0)			2.6 (1.8–3.6)			12.9 (7.5–21.9)	
Occupation									
Clinical	103	13.4 (8.9–20.1)	0.14		2.6 (2.1–3.3)	0.80		21.1 (16.3–27.3)	0.004
Nonclinical	23	6.6 (2.6–16.6)			2.4 (1.5–4.0)			8.2 (4.0–17.0)	

*Msg, major surface glycoprotein; GM, geometric mean; CI, confidence interval; Ref, reference category; PCP, *P.* *jirovecii* pneumonia. Serum antibody levels were calculated from standard curves derived by using a standard serum pool with an assigned value of 100 U; specimens below the curve were assigned the lowest possible value (1 U). Predictor variables were compared with the natural log of Msg levels using Student *t* test, then converted back to the original scale and presented as GM. Participants in this cross-sectional study were healthcare staff recruited during January 2007–February 2008 from San Francisco General Hospital.

Antibody levels to MsgA or MsgB did not differ significantly by age, sex, race, ethnicity, smoking status, presence of chronic lung disease, or presence of immunocompromising condition (Table 2). Similarly, MsgA or MsgB levels did not differ significantly between participants with and without exposure to a PCP patient or between the clinical and nonclinical groups.

In contrast, antibody levels to MsgC1 differed significantly (Table 2). Participants >60 years of age had significantly lower GM antibody levels than all others (4.8 vs. 19.1, p = 0.03); Asians had higher GM antibody levels than non-Asians (27.5 vs. 15.3, p = 0.05); and men had nonsignificantly higher GM antibody levels than women (22.7 vs. 14.8, p = 0.1). In contrast to the findings for MsgA and MsgB, participants in the clinical occupation group had significantly higher GM MsgC1 antibody levels than those in the nonclinical occupation group (21.1 vs. 8.2, p = 0.004).

### Predictors of MsgC1 Antibody Levels

To identify independent predictors of antibody levels to MsgC1, we performed multivariate linear regression and included in the model variables that had p<0.1 in bivariate analysis: older age, Asian race, sex, and clinical occupation. Clinical occupation (p = 0.003) and age >60 years (p = 0.03) were the only significant independent predictors of MsgC1 antibody response when we controlled for other factors (Table 3). Because the proportion of persons with immunocompromising conditions differed between the clinical and nonclinical groups, we included this variable in a subsequent model and found that it strengthened the association between clinical occupation and MsgC1 levels (coefficient 0.98, 95% CI 0.38–1.58, p = 0.001).

**Table 3:** Factors associated with MsgC1 levels in a multivariate linear regression analysis*

Factor	Estimate (95% CI)	p value
Age >60 y	−1.34 (−2.51 to −0.16)	0.03
Male sex	0.44 (−0.03 to 0.91)	0.07
Asian race	0.42 (−0.12 to 0.95)	0.13
Clinical occupation	0.89 (0.29 to 1.48)	0.003
F value	5.15	<0.001
R*^2^*	0.15	

*Msg, major surface glycoprotein; CI, confidence interval. A multivariate linear regression was generated by using the natural log of Msg levels as the dependent variable and including those predictor variables with p<0.10 in bivariate analysis. Participants in this cross-sectional study were healthcare staff recruited during January 2007–February 2008 from San Francisco General Hospital.

**Figure 2:**
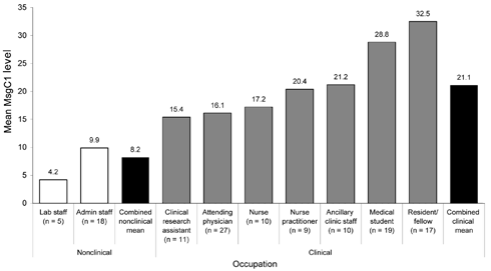
Major surface glycoprotein C1 (MsgC1) levels by occupation. Geometric mean MsgC1 levels are shown for nonclinical and clinical staff, by job title.

The GM MsgC1 antibody level was significantly higher in the clinical than in the nonclinical occupation group. Within the clinical group, we examined the association between direct patient care occupations and indirect patient care occupations and MsgC1 antibody levels. We found no significant difference in GM MsgC1 antibody levels between these 2 groups (21.8 for direct care group vs. 18.8 for indirect care group, p = 0.64). Finally, when we examined the association between individual occupations and MsgC1 antibody levels (Figure 2), medical students (28.8) and residents/fellows (32.5) had the highest GM MsgC1 antibody levels of all participants. Laboratory staff had the lowest GM levels (4.2), but these levels did not differ significantly from those of administrative staff (9.9) (p = 0.33).

## Discussion

We investigated whether HCW occupation was associated with immune response to *P. jirovecii*. Staff in the clinical occupation group had significantly higher antibody levels to MsgC1 than did those in the nonclinical occupation group, and clinical occupation was the most significant predictor of MsgC1 antibody levels. In contrast, HCW occupation was not significantly associated with antibody levels to either MsgA or MsgB.

Why did we find an association between occupation and MsgC1 but not MsgA or MsgB antibody levels? MsgC1 is the most conserved of the 3 Msg fragments studied (*36*). When a person is repeatedly exposed to *P. jirovecii*, MsgC1 possibly acts as a recall antigen and elicits higher amnestic responses than primary antigens, such as MsgA and MsgB. Previously, we found a significant association between HIV-infected patients with PCP and changes in MsgC1 antibody levels but not with MsgA or MsgB antibody levels (*37*). In PCP patients, antibody levels of MsgC1, but not MsgA or MsgB, increased significantly from baseline to weeks 3–4 (after treatment completion). Furthermore, MsgC1 antibody levels increased only in patients with PCP; levels remained unchanged in patients with non-*P. jirovecii* pneumonia. Thus, increases in MsgC1 levels may indicate recovery from or recent exposure to PCP, and this assay may be a useful epidemiologic tool for studying *P.* *jirovecii* infection.

The MsgC1 assay may also be useful in the study of *P.* *jirovecii* exposure and colonization. In the laboratory, *Pneumocystis* colonizes immunocompetent animals after they are exposed to either animals with *Pneumocystis* pneumonia or animals colonized with *Pneumocystis* (*22*,*23*). Colonized animals remain without evidence of pneumonia but can serve as a source of *Pneumocystis* because *Pneumocystis* pneumonia develops in immunocompromised animals subsequently placed in contact with them. These findings suggest that HCWs exposed to patients with PCP (or persons colonized with *P. jirovecii*) can become colonized with *P. jirovecii* and that the MsgC1 ELISA might be used as an indication of this colonization, although the precise relation between antibody levels and *P. jirovecii* colonization has yet to be fully determined.

Prior studies of *P. jirovecii* exposure and colonization in HCWs have yielded mixed results. Girón et al. observed that antibody levels in intensive care unit staff rose after exposure to a PCP patient, whereas those of control staff did not (*25*). Leigh et al. found that antibody levels of HCWs caring for patients with AIDS were significantly higher than those in control workers caring for elderly patients (*26*). Vargas et al. detected *P. jirovecii* DNA in deep nasal swab samples of 2 HCW contacts and 1 family contact of a PCP patient, compared with no detectable DNA in control HCWs who did not enter the patient’s room (*29*). Likewise, Miller et al. demonstrated that HCWs in an HIV/AIDS unit were more likely to have detectable *P. jirovecii* DNA in induced sputum and nasal rinse samples than were those working on a general medical–respiratory unit (*30*). Durand-Joly et al. longitudinally followed HCWs with oropharyngeal washes and found *P. jirovecii* DNA in 10 (6.1%) of 164; duration of carriage ranged from 3 to 10 weeks (*31*).

In contrast, other studies found no evidence of *P. jirovecii* colonization in HCWs. Lundgren et al. found no difference in the frequency or level of *P. jirovecii* Msg antibodies between HCWs caring for PCP patients in an infectious diseases department and control staff from a blood bank and surgical department (*28*). In addition, they were unable to detect *P. jirovecii* DNA in oropharyngeal washes. Similarly, Lidman and colleagues found no evidence of *P. jirovecii* DNA in sputum samples from nurses caring for a PCP patient, and only 2 (8%) of 26 had detectable antibodies to *P. jirovecii* (*27*). The conflicting results from these studies may be due to several factors, including differences in types of staff recruited, specimens collected, or assays used. Staff working in different clinical departments are likely to have differing degrees of exposure to PCP patients. For example, HCWs working on an HIV/AIDS unit (Miller et al. study) may have cared for more PCP patients than did those working in a general infectious diseases department (Lundgren et al. study). This difference in *P. jirovecii* exposure may have contributed to positive findings in the first study and negative findings in the second. In addition, different respiratory specimens have different degrees of invasiveness and different organismal yields, ranging from more invasive bronchoalveolar lavage to less invasive oropharyngeal washes. Similarly, different assays have varying sensitivities for detecting the organism. Among the DNA-based assays, nested PCR is more sensitive than single-round PCR for detecting *P. jirovecii* DNA. For serum assays, ELISA may be more sensitive but Western blot is more specific. Prevalence of the organism also may differ in different geographic regions, as has been seen in various European populations (*4*,*38*,*39*).

Our observation of higher MsgC1 antibody levels in clinical staff than in nonclinical staff is consistent with the possibility of nosocomial transmission of *P. jirovecii*. Moreover, immunocompetent HCWs may serve as reservoirs. *P. jirovecii* may circulate among patients and staff in the hospital, causing colonization in immunocompetent staff and pneumonia in immunocompromised patients. Studies in laboratory animals and outbreaks of PCP within clinical settings support this theory and person-to-person transmission as the mode of transmission. If this pattern does indeed occur, it would have clinically relevant implications for hospital infection control policies.

Our study is limited by an imprecise measure of *P. jirovecii* exposure. Most staff can recall direct contact with PCP patients but cannot recall transient exposure or indirect contact. In addition, asymptomatic persons colonized with *P. jirovecii* may be another source of exposure not recognized by HCWs. Our cross-sectional study design and method of data collection (participant recall) limit our ability to accurately capture information about *P. jirovecii* exposure and intensity of exposure over time. Comparing hospital staff with patient contact to those without patient contact allowed us to evaluate whether clinical occupation was independently associated with *P. jirovecii* antibody levels. Although the nonclinical group had a higher proportion of immunocompromising conditions than did the clinical group, this difference most likely did not influence the results. Whether an immunocompromising condition would be associated with lower antibody levels caused by impaired antibody production or higher antibody levels caused by permissive colonization is unclear. Our results suggest that the presence of an immunocompromising condition may be associated with higher levels. Regardless, including this predictor in the multivariate analysis only strengthened the association of clinical occupation and MsgC1 levels. Norris and colleagues showed that a rise in antibody levels is associated with colonization in nonhuman primates (*40*), and although antibody levels may not correlate directly with colonization, a similar pattern might occur in humans.

Early serologic studies of *P. jirovecii* led investigators to conclude that PCP results from reactivation of latent infection; however, our data add to more recent studies suggesting that PCP may be acquired from recent exposure. Higher antibody levels in clinical staff than in nonclinical staff may be evidence of patient-to-provider transmission of *P. jirovecii*. Furthermore, evidence from animal models suggests that parallel provider-to-patient transmission could occur with immunocompromised patients. Future studies should use serologic assays, such as the MsgC1 assay, as epidemiologic tools to assess *P. jirovecii* exposure in HCWs longitudinally, in addition to examining direct measures of colonization such as *P. jirovecii* DNA in respiratory specimens. If further data support the pattern observed here, clinicians may consider respiratory precautions and respiratory isolation when caring for patients with PCP. Preventing *P. jirovecii* transmission may further decrease the incidence of this grave disease.
